# The efficacy of stereotactic radiotherapy followed by bevacizumab and temozolomide in the treatment of recurrent glioblastoma: a case report

**DOI:** 10.3389/fphar.2024.1401000

**Published:** 2024-09-04

**Authors:** Wangyan Zhong, Jiwei Mao, Dongping Wu, Jianghua Peng, Wanli Ye

**Affiliations:** ^1^ Department of Radiation Oncology, Shaoxing People’s Hospital, Shaoxing, Zhejiang, China; ^2^ Department of General Practice, Shaoxing People’s Hospital, Shaoxing, Zhejiang, China

**Keywords:** stereotactic radiotherapy, bevacizumab, temozolomide, recurrent glioblastoma, combination therapy

## Abstract

Glioblastoma (GBM) is the most common and aggressive malignant brain tumor among adults. Despite advancements in multimodality therapy for GBM, the overall prognosis remains poor, with an extremely high risk of recurrence. Currently, there is no established consensus on the optimal treatment option for recurrent GBM, which may include reoperation, reirradiation, chemotherapy, or a combination of the above. Bevacizumab is considered a first-line treatment option for recurrent GBM, as is temozolomide. However, in recurrent GBM, it is necessary to balance the risks and benefits of reirradiation in combination with bevacizumab and temozolomide. Herein, we report the case of a patient with recurrent GBM after standard treatment who benefited from stereotactic radiotherapy followed by bevacizumab and temozolomide maintenance therapy. Following 16 months of concurrent chemoradiotherapy (CCRT), the patient was diagnosed with recurrent GBM by a 3-T contrast-enhanced magnetic resonance imaging (MRI). The addition of localized radiotherapy to the ongoing treatment regimen of bevacizumab, in combination with temozolomide therapy, prolonged the patient’s disease-free survival to over 2 years, achieving a significant long-term outcome, with no notable adverse effects observed. This clinical case may provide a promising new option for patients with recurrent GBM.

## Introduction

Glioblastoma (GBM, World Health Organization grade IV) is the most common and aggressive malignant brain tumor among adults, representing about 14.5% of all brain tumor (Pirozzi et al., 2022). The incidence rate of GBM is roughly 3–4 cases per 100,000 individuals annually, with a higher prevalence in those older than 65 years old (Vaz-Salgado et al., 2023). According to the National Comprehensive Cancer Network (NCCN) guidelines, maximally safe resection, followed by chemoradiotherapy with temozolomide, and subsequent administration of adjuvant temozolomide monotherapy for 6 cycles is the standard of care for newly diagnosed GBM (Horbinski et al., 2023). In recent years, The combination therapy of Tumor Treating Fields (TTFields) with temozolomide extended median progression-free survival (PFS) to 7.1 months and median overall survival (OS) to 20.5 months, in contrast to the temozolomide monotherapy group, which had median PFS of 4.0 months and median OS of 15.6 months. (Stupp et al., 2015). Based on these results, TTFields was approved by the Food and Drug Administration(FDA) for patients with newly diagnosed GBM in 2015 (Ali et al., 2022).

Despite advancements in multimodality therapy for GBM, the overall prognosis remains poor, with an average OS of 12–15 months (Stupp et al., 2009; Ghosh et al., 2018). Approximately 90% of patients experience recurrence following standard-of-care treatment, within a median interval of 8 months (Minniti et al., 2021). Patients with recurrent GBM only have a median OS of less than 6 months. Currently, there is no established consensus on the optimal treatment option for recurrent GBM. Reirradiation is increasingly used in the management of recurrent GBM, however, recurrence often occurs within or adjacent to the original tumor site, which may result in radiation necrosis (Knisely and Fine, 2023). In recent years, technological advances in radiation have reduced the risk of toxicity, making reirradiation as a feasible treatment option (Scaringi et al., 2018). As GBM is a hyperemic tumor with high expression of VEGF (Seyedmirzaei et al., 2021), VEGFA is a reasonable target molecule in the treatment of GBM. Bevacizumab (BEV), a humanized monoclonal antibody inhibiting VEGFA and the most commonly used antiangiogenic agent, has shown promising results in reducing side effects from the radiation (Kamiya-Matsuoka and Gilbert, 2015; Larson et al., 2014)and improving the quality of life (Weller et al., 2021). NRG Oncology/RTOG1205 showed that the combination of bevacizumab with reirradiation *versus* bevacizumab monotherapy as treatment for recurrent GBM confirmed meaningful improvement in PFS, although no significant difference in OS was observed (Tsien et al., 2023). A retrospective study indicated that the combination of re-irradiation with temozolomide for the treatment of recurrent GBM showed superior efficacy in extending the median OS and PFS compared to re-irradiation combined with bevacizumab (17 months *versus* 12 months for OS; 6.6 months *versus* 5.1 months for PFS) as well as nitrosoureas (6.6 months *versus* 2.9 months for PFS; 17 months *versus* 8 months for OS). (Baehr et al., 2020). However, whether stereotactic radiotherapy (SRT) followed by maintenance therapy with bevacizumab and temozolomide can effectively treat patients with recurrent GBM remains unknown. In the case study of recurrent GBM that we have documented, the patient exhibited a prolonged disease-free survival interval exceeding 24 months, which was attributed to a multimodal therapeutic approach that included the concomitant administration of temozolomide and bevacizumab, complemented by focal radiotherapy. This clinical outcome contributes a significant insight into the potential efficacy of this treatment combination as a therapeutic modality for patients with recurrent GBM, thereby warranting further investigation and consideration within the clinical setting.

## Clinical presentation

**FIGURE 1:**
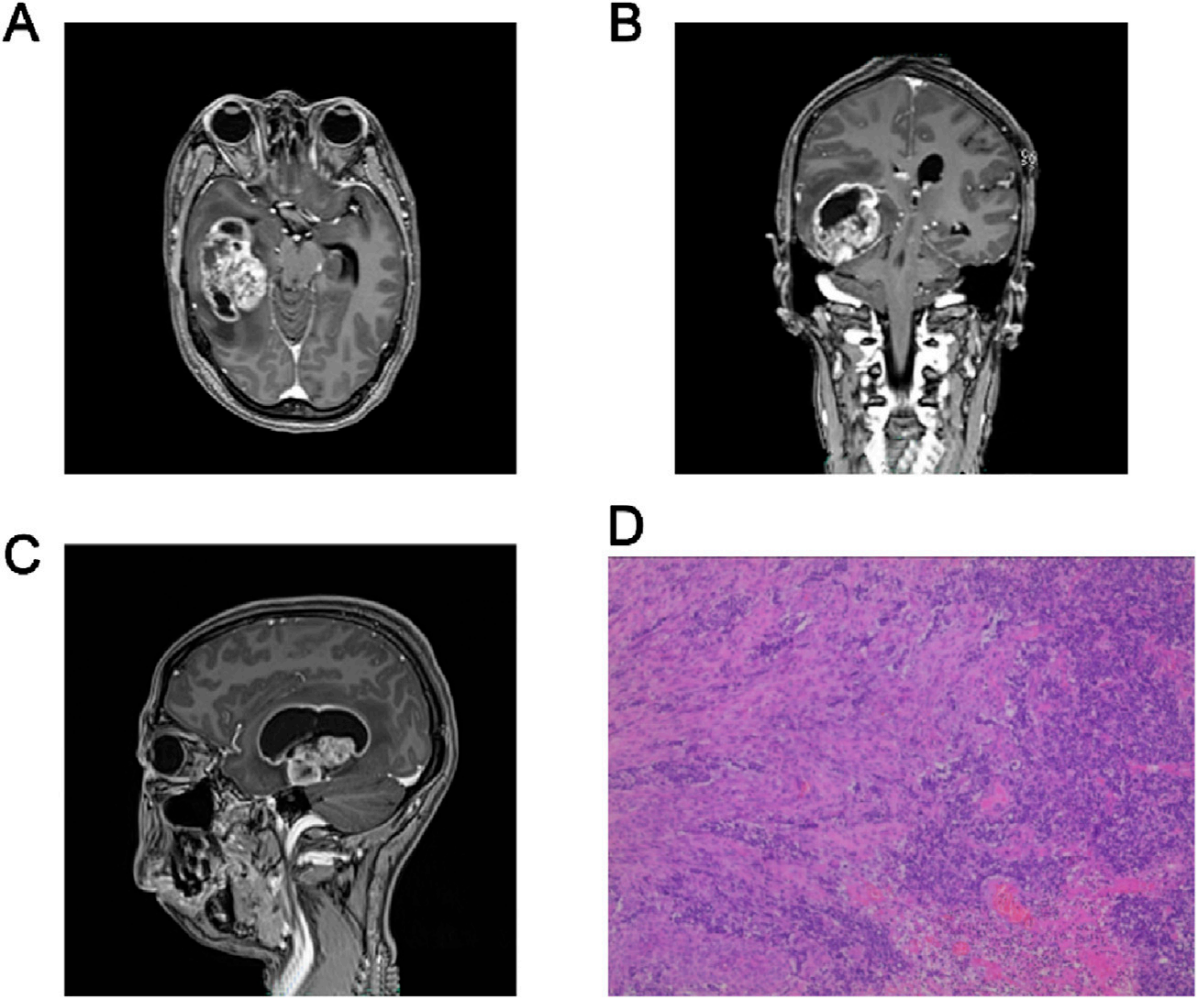
Diagnosis of Glioblastoma. Axial **(A)**, coronal **(B)** and sagittal **(C)** Diagnostic imaging result. T1-weighted MRI demonstrating a contrast-rim-enhancing lesion of the right temporal lobe. **(D)** Hematoxylin and eosin stains of the tumor, showing vascular endothelial cell proliferation at 100x.

**FIGURE 2:**
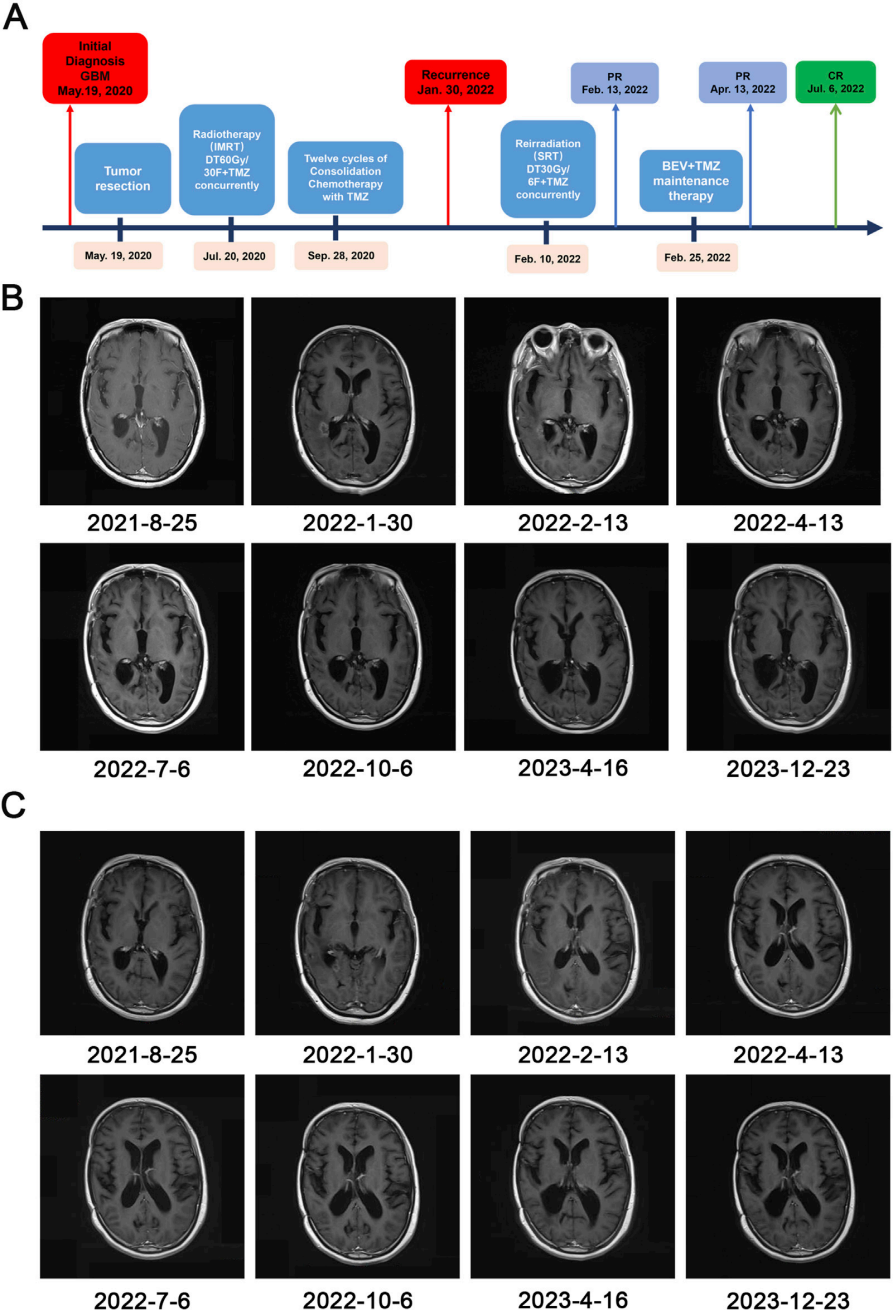
Timeline of treatment and radiographic responses. **(A)** Timeline of treatment. Abbreviations: Glioblastoma (GBM), partial response (PR), complete response (CR), bevacizumab (BEV), temozolomide (TMZ), stereotactic radiotherapy (SRT); **(B, C)** The post-contrast T1-weighted MRI showing regression of the lesion and demonstrating the effectiveness of SRT followed by bevacizumab (BEV) and temozolomide (TMZ).

A previously healthy 47-year-old woman was admitted to Shaoxing People’s Hospital in the middle of March 2020 with a history of headaches for 7 days. The patient suddenly developed a severe headache with nausea and vomiting of stomach contents, without a known cause. The physical examination yielded no abnormal or pathologic signs. A computed tomography (CT) scan revealed a neoplastic lesion located in the right temporal lobe. The administration of mannitol, targeting the reduction of intracranial hypertension, resulted in the alleviation of headache symptoms. The patient denies a history of diabetes mellitus, hypertension, previous surgical procedures, trauma, and a family history of cancer. A 3-T contrast-enhanced magnetic resonance imaging (MRI) scans of the brain identified an irregular mixed cystic and solid mass in the right temporal lobe. Post-contrast imaging revealed intense enhancement of the solid portions of the lesion. There was appreciable compression of the adjacent brain parenchyma, with concomitant displacement of the intracranial midline structures to the left (Figures 1A–C). On 19 May 2020, the patient underwent right temporal craniotomy for gross total resection at our hospital. The surgical intervention was conducted without incident, and the patient exhibited satisfactory postoperative recovery, devoid of neurological impairment. Subsequent cranial CT demonstrated postoperative cerebral edema localized to the surgical region, which was successfully mitigated with the administration of osmotic diuretic therapy, specifically mannitol. Postoperative pathology and genetic examination confirmed the diagnosis of glioblastoma [World Health Organization (WHO) stage IV] (Figure 1D), with an IDH wild-type genotype and a positive methylation status of the MGMT promoter. Then, postoperative adjuvant radiotherapy (60 Gy in 30 fractions) and temozolomide chemotherapy (75 mg/m^2^, qd, days 1–42) concurrently were given on 20 July 2020 (Figure 2A). Subsequently, twelve cycles of consolidation temozolomide chemotherapy (150–200 mg/m^2^, qd, days 1–5, every 28 days (q28d)) were performed. Subsequently, the patient was scheduled for a 3-T contrast-enhanced MRI at approximately quarterly intervals, and no signs of tumor recurrence were detected during the follow-up period. However, on 30 January 2022, a 3-T MRI scan identified two novel abnormal signal intensities adjacent to the posterolateral horns of the right lateral ventricle. Post-contrast imaging revealed these lesions to exhibit pronounced and irregular “ring-enhancement” patterns, which are suggestive of tumor recurrence (Figures 2B,C dated 2022.01.30).

Based on the above, 16 months after the CCRT, the patient was diagnosed with recurrent GBM by craniocerebral MRI. Thereafter, the patient received stereotactic radiotherapy (SRT) (30 Gy in 6 fractions). Maintenance treatment with bevacizumab (300 mg, every 2 weeks) combined with temozolomide (320 mg, qd, days 1–5, every 28 days) was initiated after the completion of SRT. Craniocerebral MRI scans demonstrated a further reduction in tumor foci size, culminating in a final response evaluation of complete response (CR) (Figure 2B 2022.07.6). The patient experienced mild hepatic impairment after 4 months of combined therapy and grade 2 hypertension after 5 months. The patient has shown an improvement trend in liver function indicators following symptomatic treatment with polyene phosphatidylcholine for liver dysfunction. Concurrently, the blood pressure levels have been effectively controlled with the administration of irbesartan and hydrochlorothiazide tablets for antihypertensive therapy. As of 1 March 2024, the patient, in addition to hypertension and impaired liver function, experiences intermittent episodes of nausea and vomiting, without any other significant complications or adverse reactions. Based on this, the treatment regimen of bevacizumab in combination with temozolomide for maintenance therapy continues to be administered.

## Discussion

This study explored the efficacy of SRT followed by bevacizumab and temozolomide as a treatment strategy for patients experiencing first recurrent GBM after standard care, which includes surgery, CCRT and consolidation chemotherapy after postoperative adjuvant radiotherapy. The optimal treatment option for recurrent GBM is controversial, which may include reoperation, reirradiation, chemotherapy, or a combination of the above (Weller et al., 2013; Seystahl et al., 2016). Repeat surgical management increases the risk of regional complications, such as wound infection and cerebrospinal fluid(CSF) leak, particularly in patients who received radiation treatment (Hoover et al., 2013). It has been suggested that repeat resection is a risk factor for complication and demonstrate no improvement in terms of survival or quality of life (Goldman et al., 2018). Technological advances in radiation, such as stereotactic radiosurgery (SRS) and stereotactic radiotherapy (SRT), have improved the therapeutic ratio (median overall survival of 6–12 months) with tolerable neurological toxicity (Minniti et al., 2021; Scaringi et al., 2018; Kazmi et al., 2019). Consequently, reirradiation was recommended by international guideline for young patients with good Karnofsky Performance Status (KPS) facing recurrent GBM, especially after a long period of interval from prior radiation (Sulman et al., 2017). Nevertheless, recurrent GBM remains an enormous therapeutic challenge duo to a very poor prognosis (Clarke et al., 2010). It is urgent to identify a consolidation therapy after local reirradiation to improve the prognosis for patients with recurrent GBM.

Tumor angiogenesis plays a significant role in the tumorigenesis, migration, and growth of solid tumors such as GBM, involving the upregulation of proangiogenic growth factors, particularly vascular endothelial growth factor (VEGF) (Seyedmirzaei et al., 2021), which indicates the potential value of treatments targeting tumor vasculature (McGee et al., 2010). Bevacizumab is a broad-spectrum anticancer drug engineered to inhibit VEGF, thereby it inhibits the formation of tumor neovascularization (Fu et al., 2023). Recently, Bevacizumab has been increasingly used in recurrent GBM as a combination treatment with immunotherapy (Chiu et al., 2023), radiotherapy (Tsien et al., 2023; Kulinich et al., 2021), and chemotherapy (Taal et al., 2014; Sepúlveda et al., 2015). In a clinical trial evaluating the combination therapy of bevacizumab with temozolomide in patients experiencing recurrence of GBM, the median progression-free survival (PFS) and overall survival (OS) were determined to be 9.5 months and 15.4 months, respectively (Sepúlveda et al., 2015). The NRG Oncology/RTOG1205 trial demonstrated enhanced PFS and higher 6-month PFS rates for patients with recurrent GBM in the bevacizumab combined with re-irradiation therapy group compared to those treated with bevacizumab alone, reporting median PFS durations of 7.1 and 3.8 months, respectively (Tsien et al., 2023). These studies have shown survival benefit and safety profiles for bevacizumab (Tsien et al., 2023; Sepúlveda et al., 2015). Based on these results, bevacizumab was included in the list of medicare funded by the National Health Service for the treatment of the recurrent GBM in 2022 in China. Temozolomide, an oral DNA alkylating agent, represents a common first-line therapeutic option for recurrent GBM, especially with a positive methylation status of the MGMT promoter (Minniti et al., 2021; Balañá et al., 2011; Rodríguez-Camacho et al., 2022). However, whether reirradiation followed by maintenance therapy with bevacizumab and temozolomide can effectively treat patients with recurrent GBM remains unknown. A randomized prospective trial has delineated a substantial therapeutic advantage, wherein the integration of reirradiation with bevacizumab-combined chemotherapy significantly extends PFS for patients suffering from recurrent bevacizumab-resistant high-grade gliomas, in contrast to bevacizumab-combined chemotherapy alone (5.1 months *versus* 1.8 months; *p* < 0.001) (Bergman et al., 2020). The results imply that the integration of reirradiation with bevacizumab-combined chemotherapy significantly extends PFS for patients of recurrent bevacizumab-resistant GBM. These findings suggest that the patients with first recurrent GBM may benefit from SRT followed by a combination of bevacizumab plus temozolomide, which is likely to be effective and tolerable.

In a phase II study by Refae et al., 59 patients of GBM were randomized to receive either 6 cycles or more than 6 cycles of adjuvant temozolomide. Both OS and PFS were significantly better in the group receiving a longer temozolomide duration (Median PFS: 12.1 months for 6 cycles vs 18.8 months for >6 cycles; *p* = 0.015. Median OS: 18.1 months for 6 cycles vs 24.1 months for >6 cycles; *p* = 0.048) (<1eb28fdb5da24f8d93da550609bead37. pdf>.). Similar studies advocate prolonged use of adjuvant temozolomide for more than 6 cycle (Alimohammadi et al., 2020). In this case, twelve cycles of consolidation temozolomide chemotherapy were administered subsequent to postoperative adjuvant radiotherapy. The patient experienced a recurrence 20 months after surgery (a median time interval of 8 months). According to the NCCN guidelines, repeat surgery or reirradiation is the preferred strategy for managing recurrent GBM. However, for the recurrent tumor located in the right lateral ventricle, surgical intervention may lead to neurological complications and has a negative impact on patient’s quality of life. Thus, the patient refused to repeat surgery. When selecting an appropriate patient (localized recurrence, time since first irradiation >12 months) (Vaz-Salgado et al., 2023), reirradiation has been recognized as an effective and safe treatment. In select clinical investigations, patients with recurrent GBM who received high-dose hypofractionated SRT at doses of 25–35 Gy in 5–7 Gy per fraction, have demonstrated a survival time ranging from 7.3 to 12.5 months (Greenspoon et al., 2014; Minniti et al., 2012; Gutin et al., 2009; Grosu et al., 2005). Aligning with the patient’s preferences and according to previous studies, we select SRT (30 Gy in 6 fractions). The patient was in generally good condition without signs of radiation necrosis or significant neurological deficits. Given that local radiotherapy only extended median overall survival by 7.3–12.5months, how to further improve the outcome is crucial. A single-center retrospective study of patients with recurrent GBM treated with bevacizumab found that the median PFS in the low-dose regimen(5 mg/kg every 2–3 weeks or 10 mg/kg every 3 weeks) was 5.89 months (95% CI 3.72–7.5 months), compared to a median PFS of 3.22 months (95% CI 2.27–4.70 months) in the standard-dose regimen((10 mg/kg every 2 weeks) (Melhem et al., 2023). Furthermore, despite the absence of larger-scale trials, certain studies seem to imply that a reduced dosage of bevacizumab may correlate with enhanced overall survival outcomes (Levin et al., 2015; Ajlan et al., 2017). Therefore, in this case, the patient was administered bevacizumab at half the standard dose (5 mg/kg, every 2 weeks) and temozolomide (200 mg/m2 body surface/day, 150 mg/m2 body surface/day for the first time) on days 1–5, every 28 days following the completion of SRT. The patient responded well to this combination therapy, with a dramatical reduction in lesion size. Though the patient developed mild hepatic impairment after 4 months of combined therapy and grade 2 hypertension after 5 months, all adverse effects were managed with symptomatic treatment without dose reduction. After 4 months of combined treatment with bevacizumab and temozolomide, the clinical efficacy of the patient was evaluated as CR on 6 July 2022. By March 2024, the effect was stable with no signs of recurrence on imaging. No late toxicity was observed in this patient. In conclusion, this patient has a PFS of more than 2 years after recurrence, which has significantly exceeded the median survival time reported in the literature. The periodic follow-up will continue. This clinical case may provide a promising new option for patients with recurrent GBM.

## Conclusion

Overall, this case underscores the potential efficacy of SRT followed by bevacizumab and temozolomide in the treatment of recurrent GBM. In this patient, SRT followed by bevacizumab and temozolomide had demonstrated remarkable efficacy on recurrent GBM. However, the testing of this regimen has been limited by factors such as the small sample size of cases. Moving forward, our objective in subsequent studies is to further investigate this therapeutic approach within a larger cohort to validate its effectiveness and generalizability.

## Data Availability

The raw data supporting the conclusions of this article will be made available by the authors, without undue reservation.
